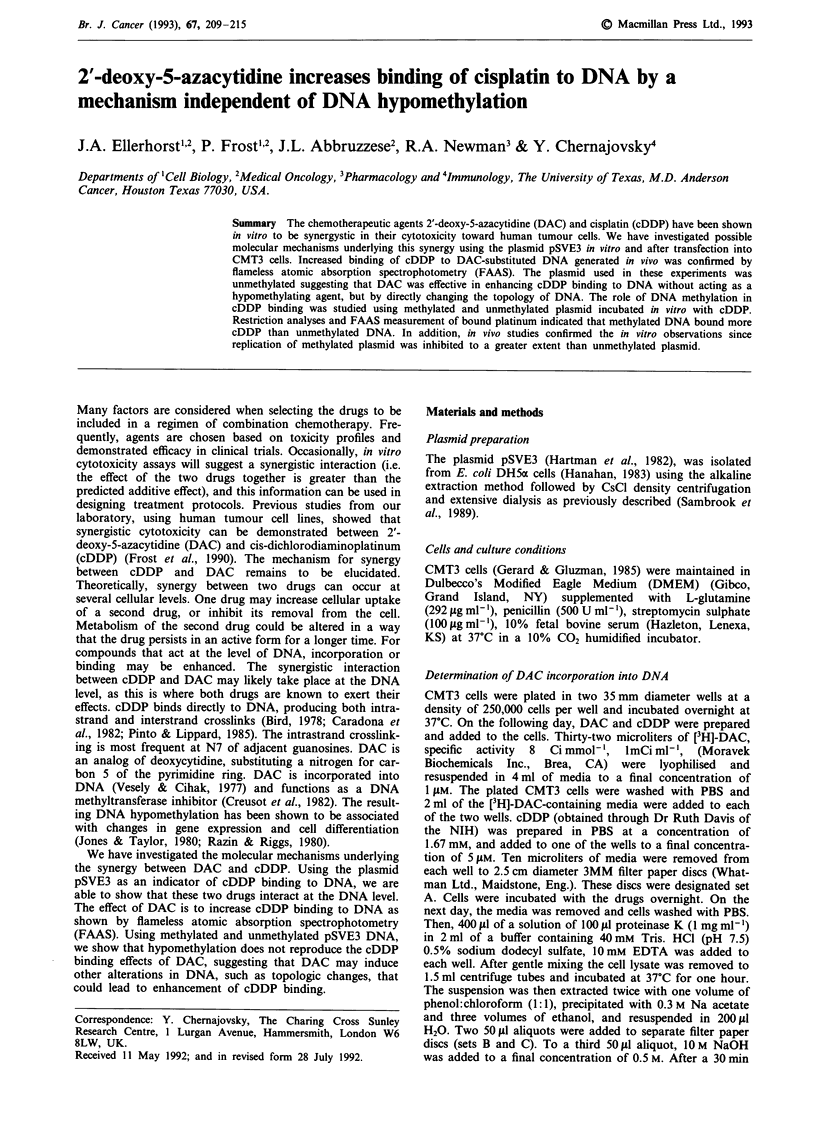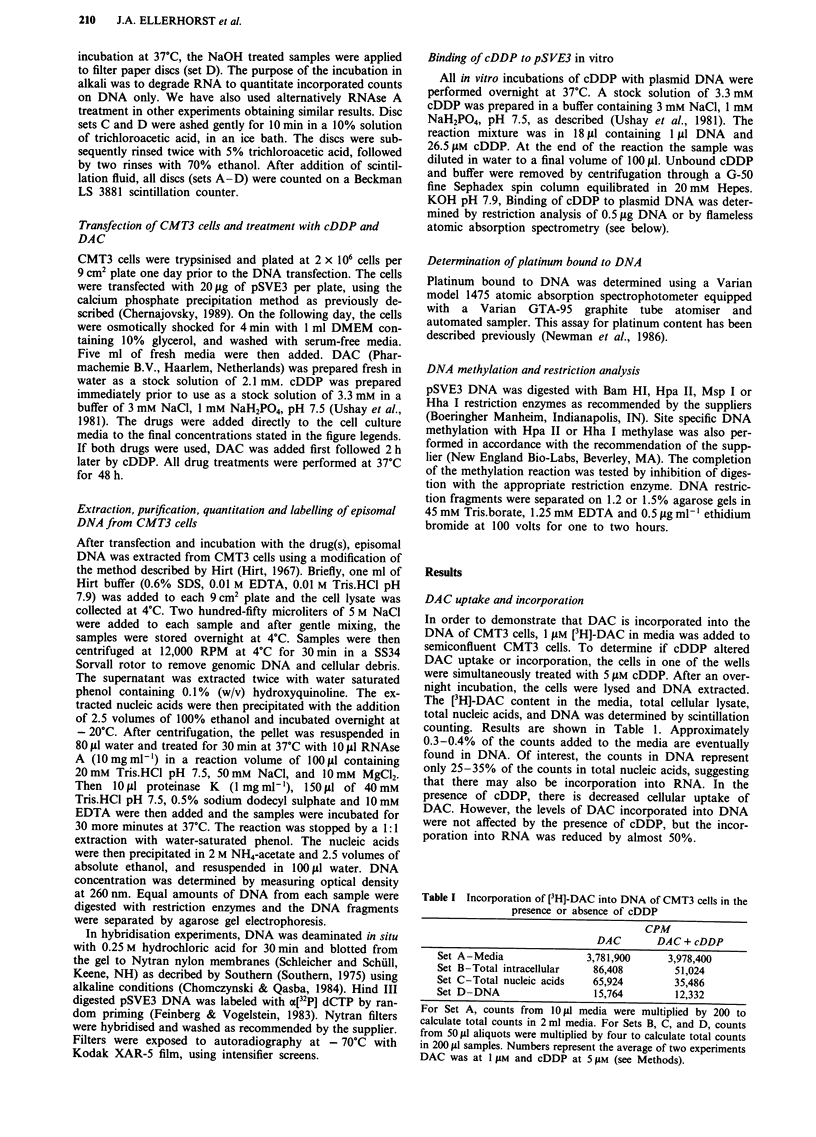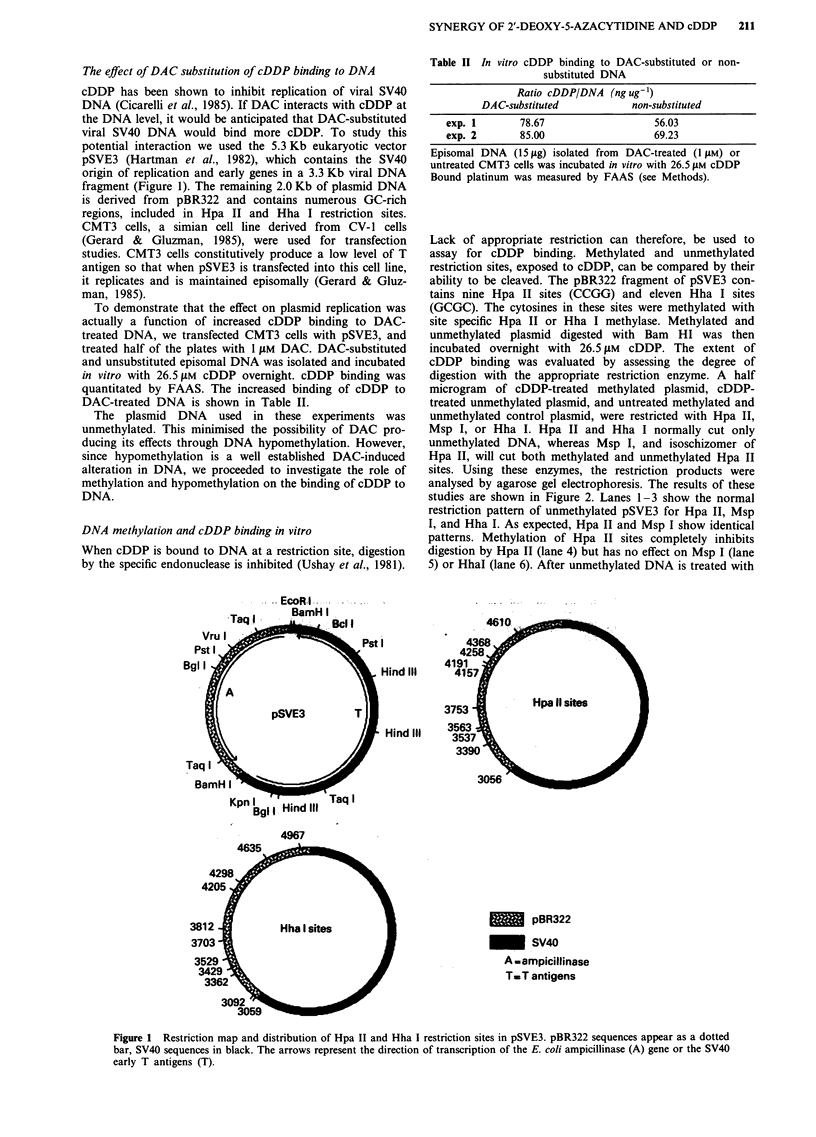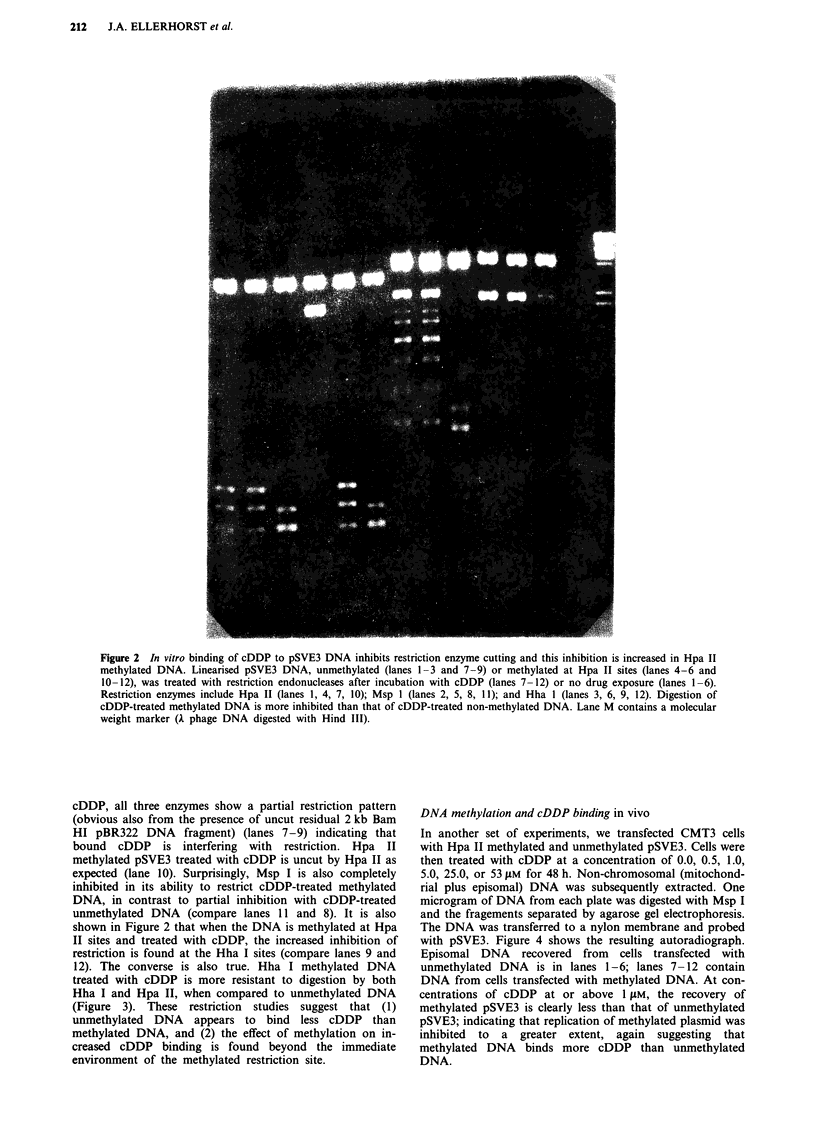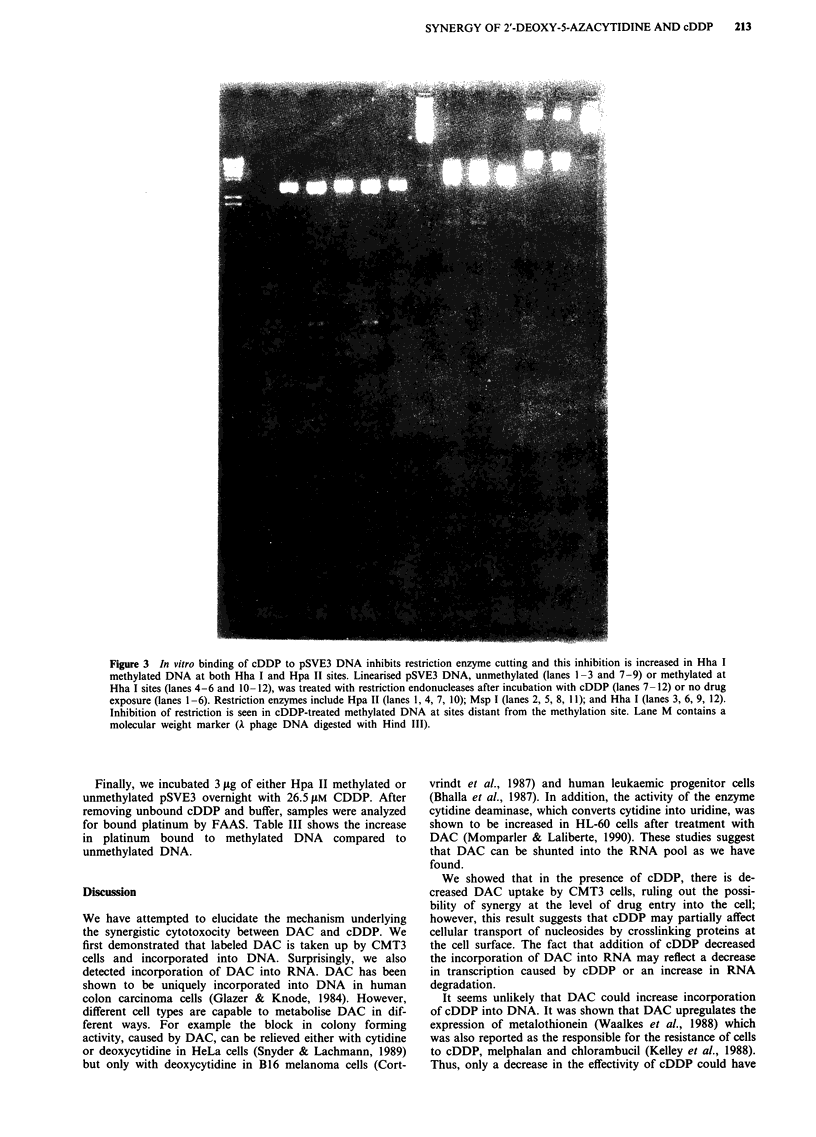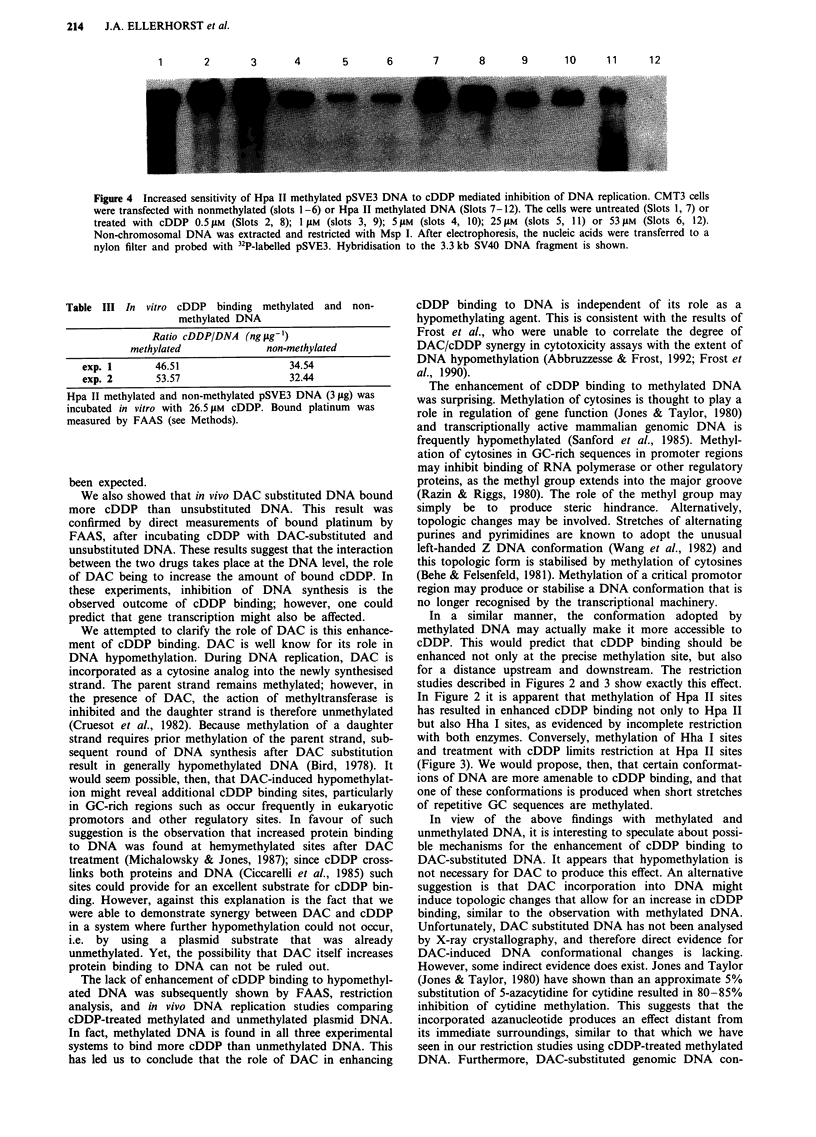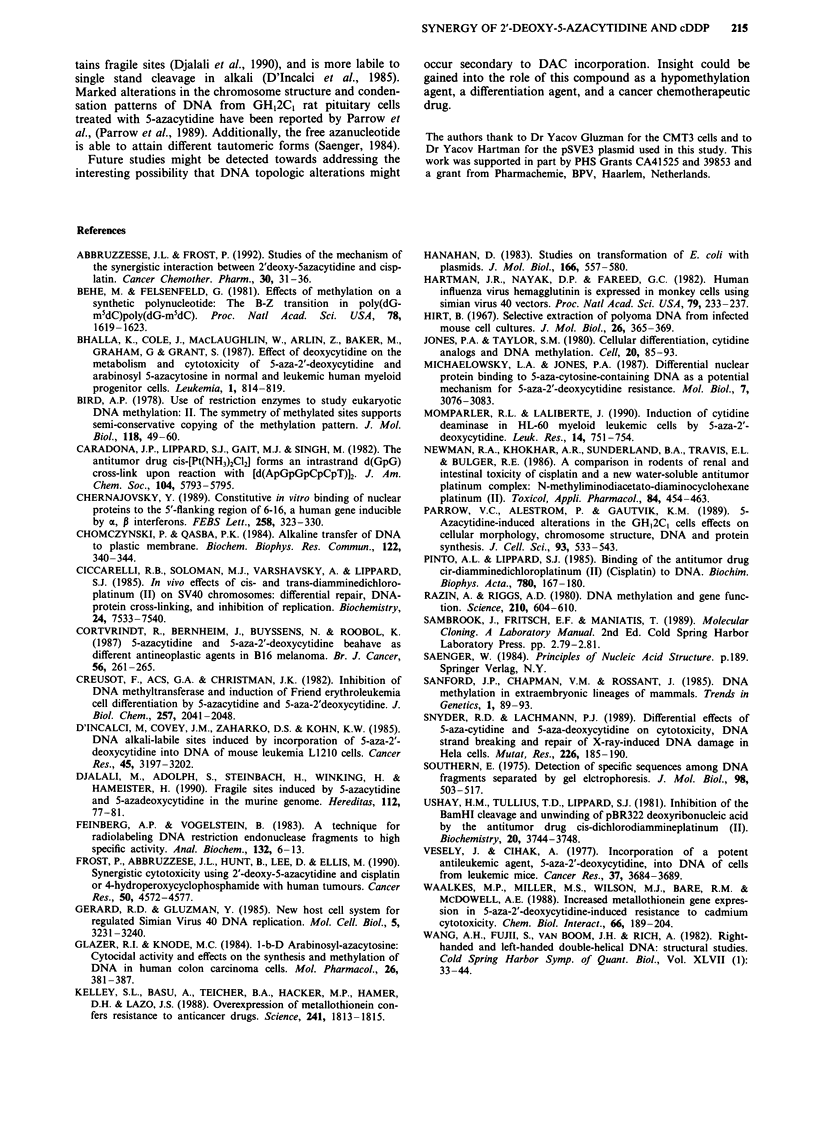# 2'-deoxy-5-azacytidine increases binding of cisplatin to DNA by a mechanism independent of DNA hypomethylation.

**DOI:** 10.1038/bjc.1993.41

**Published:** 1993-02

**Authors:** J. A. Ellerhorst, P. Frost, J. L. Abbruzzese, R. A. Newman, Y. Chernajovsky

**Affiliations:** Department of Cell Biology, University of Texas, M.D. Anderson Cancer, Houston 77030.

## Abstract

**Images:**


Br. J. Cancer (1993), 67, 209-215 © Macmillan Press Ltd., 1993

### 2′-deoxy-5-azacytidine increases binding of cisplatin to DNA by a mechanism independent of DNA hypomethylation

J.A. Ellerhorst[1,2], P. Frost[1,2], J.L. Abbruzzese[2], R.A. Newman[3] & Y. Chernajovsky[4]

*Departments of [1]Cell Biology, [2]Medical Oncology, [3]Pharmacology and [4]Immunology, The University of Texas, M.D. Anderson Cancer, Houston Texas 77030, USA.*

**Summary** The chemotherapeutic agents 2′-deoxy-5-azacytidine (DAC) and cisplatin (cDDP) have been shown *in vitro* to be synergystic in their cytotoxicity toward human tumour cells. We have investigated possible molecular mechanisms underlying this synergy using the plasmid pSVE3 *in vitro* and after transfection into CMT3 cells. Increased binding of cDDP to DAC-substituted DNA generated *in vivo* was confirmed by flameless atomic absorption spectrophotometry (FAAS). The plasmid used in these experiments was unmethylated suggesting that DAC was effective in enhancing cDDP binding to DNA without acting as a hypomethylating agent, but by directly changing the topology of DNA. The role of DNA methylation in cDDP binding was studied using methylated and unmethylated plasmid incubated *in vitro* with cDDP. Restriction analyses and FAAS measurement of bound platinum indicated that methylated DNA bound more cDDP than unmethylated DNA. In addition, *in vivo* studies confirmed the *in vitro* observations since replication of methylated plasmid was inhibited to a greater extent than unmethylated plasmid.

Many factors are considered when selecting the drugs to be included in a regimen of combination chemotherapy. Frequently, agents are chosen based on toxicity profiles and demonstrated efficacy in clinical trials. Occasionally, *in vitro* cytotoxicity assays will suggest a synergistic interaction (i.e. the effect of the two drugs together is greater than the predicted additive effect), and this information can be used in designing treatment protocols. Previous studies from our laboratory, using human tumour cell lines, showed that synergistic cytotoxicity can be demonstrated between 2′-deoxy-5-azacytidine (DAC) and cis-dichlorodiaminoplatinum (cDDP) (Frost *et al.*, 1990). The mechanism for synergy between cDDP and DAC remains to be elucidated. Theoretically, synergy between two drugs can occur at several cellular levels. One drug may increase cellular uptake of a second drug, or inhibit its removal from the cell. Metabolism of the second drug could be altered in a way that the drug persists in an active form for a longer time. For compounds that act at the level of DNA, incorporation or binding may be enhanced. The synergistic interaction between cDDP and DAC may likely take place at the DNA level, as this is where both drugs are known to exert their effects. cDDP binds directly to DNA, producing both intrastrand and interstrand crosslinks (Bird, 1978; Caradona *et al.*, 1982; Pinto & Lippard, 1985). The intrastrand crosslinking is most frequent at N7 of adjacent guanosines. DAC is an analog of deoxycytidine, substituting a nitrogen for carbon 5 of the pyrimidine ring. DAC is incorporated into DNA (Vesely & Cihak, 1977) and functions as a DNA methyltransferase inhibitor (Creusot *et al.*, 1982). The resulting DNA hypomethylation has been shown to be associated with changes in gene expression and cell differentiation (Jones & Taylor, 1980; Razin & Riggs, 1980).

We have investigated the molecular mechanisms underlying the synergy between DAC and cDDP. Using the plasmid pSVE3 as an indicator of cDDP binding to DNA, we are able to show that these two drugs interact at the DNA level. The effect of DAC is to increase cDDP binding to DNA as shown by flameless atomic absorption spectrophotometry (FAAS). Using methylated and unmethylated pSVE3 DNA, we show that hypomethylation does not reproduce the cDDP binding effects of DAC, suggesting that DAC may induce other alterations in DNA, such as topologic changes, that could lead to enhancement of cDDP binding.

Correspondence: Y. Chernajovsky, The Charing Cross Sunley Research Centre, 1 Lurgan Avenue, Hammersmith, London W6 8LW, UK.

Received 11 May 1992; and in revised form 28 July 1992.

### Materials and methods

### *Plasmid preparation*

The plasmid pSVE3 (Hartman *et al.*, 1982), was isolated from *E. coli* DH5α cells (Hanahan, 1983) using the alkaline extraction method followed by CsCl density centrifugation and extensive dialysis as previously described (Sambrook *et al.*, 1989).

### *Cells and culture conditions*

CMT3 cells (Gerard & Gluzman, 1985) were maintained in Dulbecco's Modified Eagle Medium (DMEM) (Gibco, Grand Island, NY) supplemented with L-glutamine (292 $\mu g\ ml^{-1}$), penicillin (500 $U\ ml^{-1}$), streptomycin sulphate (100 $\mu g\ ml^{-1}$), 10% fetal bovine serum (Hazleton, Lenexa, KS) at 37°C in a 10% $CO_2$ humidified incubator.

### *Determination of DAC incorporation into DNA*

CMT3 cells were plated in two 35 mm diameter wells at a density of 250,000 cells per well and incubated overnight at 37°C. On the following day, DAC and cDDP were prepared and added to the cells. Thirty-two microliters of [$^3$H]-DAC, specific activity 8 $Ci\ mmol^{-1}$, $1 mCi\ ml^{-1}$, (Moravek Biochemicals Inc., Brea, CA) were lyophilised and resuspended in 4 ml of media to a final concentration of 1 $\mu$M. The plated CMT3 cells were washed with PBS and 2 ml of the [$^3$H]-DAC-containing media were added to each of the two wells. cDDP (obtained through Dr Ruth Davis of the NIH) was prepared in PBS at a concentration of 1.67 mM, and added to one of the wells to a final concentration of 5 $\mu$M. Ten microliters of media were removed from each well to 2.5 cm diameter 3MM filter paper discs (Whatman Ltd., Maidstone, Eng.). These discs were designated set A. Cells were incubated with the drugs overnight. On the next day, the media was removed and cells washed with PBS. Then, 400 $\mu$l of a solution of 100 $\mu$l proteinase K (1 $mg\ ml^{-1}$) in 2 ml of a buffer containing 40 mM Tris. HCl (pH 7.5) 0.5% sodium dodecyl sulfate, 10 mM EDTA was added to each well. After gentle mixing the cell lysate was removed to 1.5 ml centrifuge tubes and incubated at 37°C for one hour. The suspension was then extracted twice with one volume of phenol:chloroform (1:1), precipitated with 0.3 M Na acetate and three volumes of ethanol, and resuspended in 200 $\mu$l $H_2O$. Two 50 $\mu$l aliquots were added to separate filter paper discs (sets B and C). To a third 50 $\mu$l aliquot, 10 M NaOH was added to a final concentration of 0.5 M. After a 30 min incubation at 37°C, the NaOH treated samples were applied to filter paper discs (set D). The purpose of the incubation in alkali was to degrade RNA to quantitate incorporated counts on DNA only. We have also used alternatively RNAse A treatment in other experiments obtaining similar results. Disc sets C and D were ashed gently for 10 min in a 10% solution of trichloroacetic acid, in an ice bath. The discs were subsequently rinsed twice with 5% trichloroacetic acid, followed by two rinses with 70% ethanol. After addition of scintillation fluid, all discs (sets A–D) were counted on a Beckman LS 3881 scintillation counter.

*Transfection of CMT3 cells and treatment with cDDP and DAC*

CMT3 cells were trypsinised and plated at $2 \times 10^6$ cells per 9 $cm^2$ plate one day prior to the DNA transfection. The cells were transfected with 20 µg of pSVE3 per plate, using the calcium phosphate precipitation method as previously described (Chernajovsky, 1989). On the following day, the cells were osmotically shocked for 4 min with 1 ml DMEM containing 10% glycerol, and washed with serum-free media. Five ml of fresh media were then added. DAC (Pharmachemie B.V., Haarlem, Netherlands) was prepared fresh in water as a stock solution of 2.1 mM. cDDP was prepared immediately prior to use as a stock solution of 3.3 mM in a buffer of 3 mM NaCl, 1 mM $NaH_2PO_4$, pH 7.5 (Ushay *et al.*, 1981). The drugs were added directly to the cell culture media to the final concentrations stated in the figure legends. If both drugs were used, DAC was added first followed 2 h later by cDDP. All drug treatments were performed at 37°C for 48 h.

*Extraction, purification, quantitation and labelling of episomal DNA from CMT3 cells*

After transfection and incubation with the drug(s), episomal DNA was extracted from CMT3 cells using a modification of the method described by Hirt (Hirt, 1967). Briefly, one ml of Hirt buffer (0.6% SDS, 0.01 M EDTA, 0.01 M Tris.HCl pH 7.9) was added to each 9 $cm^2$ plate and the cell lysate was collected at 4°C. Two hundred-fifty microliters of 5 M NaCl were added to each sample and after gentle mixing, the samples were stored overnight at 4°C. Samples were then centrifuged at 12,000 RPM at 4°C for 30 min in a SS34 Sorvall rotor to remove genomic DNA and cellular debris. The supernatant was extracted twice with water saturated phenol containing 0.1% (w/v) hydroxyquinoline. The extracted nucleic acids were then precipitated with the addition of 2.5 volumes of 100% ethanol and incubated overnight at − 20°C. After centrifugation, the pellet was resuspended in 80 µl water and treated for 30 min at 37°C with 10 µl RNAse A (10 mg $ml^{-1}$) in a reaction volume of 100 µl containing 20 mM Tris.HCl pH 7.5, 50 mM NaCl, and 10 mM $MgCl_2$. Then 10 µl proteinase K (1 mg $ml^{-1}$), 150 µl of 40 mM Tris.HCl pH 7.5, 0.5% sodium dodecyl sulphate and 10 mM EDTA were then added and the samples were incubated for 30 more minutes at 37°C. The reaction was stopped by a 1:1 extraction with water-saturated phenol. The nucleic acids were then precipitated in 2 M $NH_4$-acetate and 2.5 volumes of absolute ethanol, and resuspended in 100 µl water. DNA concentration was determined by measuring optical density at 260 nm. Equal amounts of DNA from each sample were digested with restriction enzymes and the DNA fragments were separated by agarose gel electrophoresis.

In hybridisation experiments, DNA was deaminated *in situ* with 0.25 M hydrochloric acid for 30 min and blotted from the gel to Nytran nylon membranes (Schleicher and Schüll, Keene, NH) as decribed by Southern (Southern, 1975) using alkaline conditions (Chomczynski & Qasba, 1984). Hind III digested pSVE3 DNA was labeled with $\alpha[^{32}P]$ dCTP by random priming (Feinberg & Vogelstein, 1983). Nytran filters were hybridised and washed as recommended by the supplier. Filters were exposed to autoradiography at − 70°C with Kodak XAR-5 film, using intensifier screens.

*Binding of cDDP to pSVE3* in vitro

All *in vitro* incubations of cDDP with plasmid DNA were performed overnight at 37°C. A stock solution of 3.3 mM cDDP was prepared in a buffer containing 3 mM NaCl, 1 mM $NaH_2PO_4$, pH 7.5, as described (Ushay *et al.*, 1981). The reaction mixture was in 18 µl containing 1 µl DNA and 26.5 µM cDDP. At the end of the reaction the sample was diluted in water to a final volume of 100 µl. Unbound cDDP and buffer were removed by centrifugation through a G-50 fine Sephadex spin column equilibrated in 20 mM Hepes. KOH pH 7.9, Binding of cDDP to plasmid DNA was determined by restriction analysis of 0.5 µg DNA or by flameless atomic absorption spectrometry (see below).

*Determination of platinum bound to DNA*

Platinum bound to DNA was determined using a Varian model 1475 atomic absorption spectrophotometer equipped with a Varian GTA-95 graphite tube atomiser and automated sampler. This assay for platinum content has been described previously (Newman *et al.*, 1986).

*DNA methylation and restriction analysis*

pSVE3 DNA was digested with Bam HI, Hpa II, Msp I or Hha I restriction enzymes as recommended by the suppliers (Boeringher Manheim, Indianapolis, IN). Site specific DNA methylation with Hpa II or Hha I methylase was also performed in accordance with the recommendation of the supplier (New England Bio-Labs, Beverley, MA). The completion of the methylation reaction was tested by inhibition of digestion with the appropriate restriction enzyme. DNA restriction fragments were separated on 1.2 or 1.5% agarose gels in 45 mM Tris.borate, 1.25 mM EDTA and 0.5 µg $ml^{-1}$ ethidium bromide at 100 volts for one to two hours.

### Results

*DAC uptake and incorporation*

In order to demonstrate that DAC is incorporated into the DNA of CMT3 cells, 1 µM [$^3$H]-DAC in media was added to semiconfluent CMT3 cells. To determine if cDDP altered DAC uptake or incorporation, the cells in one of the wells were simultaneously treated with 5 µM cDDP. After an overnight incubation, the cells were lysed and DNA extracted. The [$^3$H]-DAC content in the media, total cellular lysate, total nucleic acids, and DNA was determined by scintillation counting. Results are shown in Table 1. Approximately 0.3–0.4% of the counts added to the media are eventually found in DNA. Of interest, the counts in DNA represent only 25–35% of the counts in total nucleic acids, suggesting that there may also be incorporation into RNA. In the presence of cDDP, there is decreased cellular uptake of DAC. However, the levels of DAC incorporated into DNA were not affected by the presence of cDDP, but the incorporation into RNA was reduced by almost 50%.

**Table I** Incorporation of [$^3$H]-DAC into DNA of CMT3 cells in the presence or absence of cDDP

| | *CPM* | |
|---|---|---|
| | *DAC* | *DAC + cDDP* |
| Set A–Media | 3,781,900 | 3,978,400 |
| Set B–Total intracellular | 86,408 | 51,024 |
| Set C–Total nucleic acids | 65,924 | 35,486 |
| Set D–DNA | 15,764 | 12,332 |

For Set A, counts from 10 µl media were multiplied by 200 to calculate total counts in 2 ml media. For Sets B, C, and D, counts from 50 µl aliquots were multiplied by four to calculate total counts in 200 µl samples. Numbers represent the average of two experiments DAC was at 1 µM and cDDP at 5 µM (see Methods).

### *The effect of DAC substitution of cDDP binding to DNA*

cDDP has been shown to inhibit replication of viral SV40 DNA (Cicarelli *et al.*, 1985). If DAC interacts with cDDP at the DNA level, it would be anticipated that DAC-substituted viral SV40 DNA would bind more cDDP. To study this potential interaction we used the 5.3 Kb eukaryotic vector pSVE3 (Hartman *et al.*, 1982), which contains the SV40 origin of replication and early genes in a 3.3 Kb viral DNA fragment (Figure 1). The remaining 2.0 Kb of plasmid DNA is derived from pBR322 and contains numerous GC-rich regions, included in Hpa II and Hha I restriction sites. CMT3 cells, a simian cell line derived from CV-1 cells (Gerard & Gluzman, 1985), were used for transfection studies. CMT3 cells constitutively produce a low level of T antigen so that when pSVE3 is transfected into this cell line, it replicates and is maintained episomally (Gerard & Gluzman, 1985).

To demonstrate that the effect on plasmid replication was actually a function of increased cDDP binding to DAC-treated DNA, we transfected CMT3 cells with pSVE3, and treated half of the plates with 1 μM DAC. DAC-substituted and unsubstituted episomal DNA was isolated and incubated *in vitro* with 26.5 μM cDDP overnight. cDDP binding was quantitated by FAAS. The increased binding of cDDP to DAC-treated DNA is shown in Table II.

The plasmid DNA used in these experiments was unmethylated. This minimised the possibility of DAC producing its effects through DNA hypomethylation. However, since hypomethylation is a well established DAC-induced alteration in DNA, we proceeded to investigate the role of methylation and hypomethylation on the binding of cDDP to DNA.

### *DNA methylation and cDDP binding in vitro*

When cDDP is bound to DNA at a restriction site, digestion by the specific endonuclease is inhibited (Ushay *et al.*, 1981). Lack of appropriate restriction can therefore, be used to assay for cDDP binding. Methylated and unmethylated restriction sites, exposed to cDDP, can be compared by their ability to be cleaved. The pBR322 fragment of pSVE3 contains nine Hpa II sites (CCGG) and eleven Hha I sites (GCGC). The cytosines in these sites were methylated with site specific Hpa II or Hha I methylase. Methylated and unmethylated plasmid digested with Bam HI was then incubated overnight with 26.5 μM cDDP. The extent of cDDP binding was evaluated by assessing the degree of digestion with the appropriate restriction enzyme. A half microgram of cDDP-treated methylated plasmid, cDDP-treated unmethylated plasmid, and untreated methylated and unmethylated control plasmid, were restricted with Hpa II, Msp I, or Hha I. Hpa II and Hha I normally cut only unmethylated DNA, whereas Msp I, and isoschizomer of Hpa II, will cut both methylated and unmethylated Hpa II sites. Using these enzymes, the restriction products were analysed by agarose gel electrophoresis. The results of these studies are shown in Figure 2. Lanes 1–3 show the normal restriction pattern of unmethylated pSVE3 for Hpa II, Msp I, and Hha I. As expected, Hpa II and Msp I show identical patterns. Methylation of Hpa II sites completely inhibits digestion by Hpa II (lane 4) but has no effect on Msp I (lane 5) or HhaI (lane 6). After unmethylated DNA is treated with

**Table II** *In vitro* cDDP binding to DAC-substituted or non-substituted DNA

| | *Ratio cDDP/DNA (ng ug$^{-1}$)* | |
|---|---|---|
| | *DAC-substituted* | *non-substituted* |
| **exp. 1** | 78.67 | 56.03 |
| **exp. 2** | 85.00 | 69.23 |

Episomal DNA (15 μg) isolated from DAC-treated (1 μM) or untreated CMT3 cells was incubated *in vitro* with 26.5 μM cDDP Bound platinum was measured by FAAS (see Methods).

**Figure 1** Restriction map and distribution of Hpa II and Hha I restriction sites in pSVE3. pBR322 sequences appear as a dotted bar, SV40 sequences in black. The arrows represent the direction of transcription of the *E. coli* ampicillinase (A) gene or the SV40 early T antigens (T).

**Figure 2** *In vitro* binding of cDDP to pSVE3 DNA inhibits restriction enzyme cutting and this inhibition is increased in Hpa II methylated DNA. Linearised pSVE3 DNA, unmethylated (lanes 1–3 and 7–9) or methylated at Hpa II sites (lanes 4–6 and 10–12), was treated with restriction endonucleases after incubation with cDDP (lanes 7–12) or no drug exposure (lanes 1–6). Restriction enzymes include Hpa II (lanes 1, 4, 7, 10); Msp 1 (lanes 2, 5, 8, 11); and Hha 1 (lanes 3, 6, 9, 12). Digestion of cDDP-treated methylated DNA is more inhibited than that of cDDP-treated non-methylated DNA. Lane M contains a molecular weight marker (λ phage DNA digested with Hind III).

cDDP, all three enzymes show a partial restriction pattern (obvious also from the presence of uncut residual 2 kb Bam HI pBR322 DNA fragment) (lanes 7–9) indicating that bound cDDP is interfering with restriction. Hpa II methylated pSVE3 treated with cDDP is uncut by Hpa II as expected (lane 10). Surprisingly, Msp I is also completely inhibited in its ability to restrict cDDP-treated methylated DNA, in contrast to partial inhibition with cDDP-treated unmethylated DNA (compare lanes 11 and 8). It is also shown in Figure 2 that when the DNA is methylated at Hpa II sites and treated with cDDP, the increased inhibition of restriction is found at the Hha I sites (compare lanes 9 and 12). The converse is also true. Hha I methylated DNA treated with cDDP is more resistant to digestion by both Hha I and Hpa II, when compared to unmethylated DNA (Figure 3). These restriction studies suggest that (1) unmethylated DNA appears to bind less cDDP than methylated DNA, and (2) the effect of methylation on increased cDDP binding is found beyond the immediate environment of the methylated restriction site.

### *DNA methylation and cDDP binding* in vivo

In another set of experiments, we transfected CMT3 cells with Hpa II methylated and unmethylated pSVE3. Cells were then treated with cDDP at a concentration of 0.0, 0.5, 1.0, 5.0, 25.0, or 53 μM for 48 h. Non-chromosomal (mitochondrial plus episomal) DNA was subsequently extracted. One microgram of DNA from each plate was digested with Msp I and the fragements separated by agarose gel electrophoresis. The DNA was transferred to a nylon membrane and probed with pSVE3. Figure 4 shows the resulting autoradiograph. Episomal DNA recovered from cells transfected with unmethylated DNA is in lanes 1–6; lanes 7–12 contain DNA from cells transfected with methylated DNA. At concentrations of cDDP at or above 1 μM, the recovery of methylated pSVE3 is clearly less than that of unmethylated pSVE3; indicating that replication of methylated plasmid was inhibited to a greater extent, again suggesting that methylated DNA binds more cDDP than unmethylated DNA.

**Figure 3** *In vitro* binding of cDDP to pSVE3 DNA inhibits restriction enzyme cutting and this inhibition is increased in Hha I methylated DNA at both Hha I and Hpa II sites. Linearised pSVE3 DNA, unmethylated (lanes 1–3 and 7–9) or methylated at Hha I sites (lanes 4–6 and 10–12), was treated with restriction endonucleases after incubation with cDDP (lanes 7–12) or no drug exposure (lanes 1–6). Restriction enzymes include Hpa II (lanes 1, 4, 7, 10); Msp I (lanes 2, 5, 8, 11); and Hha I (lanes 3, 6, 9, 12). Inhibition of restriction is seen in cDDP-treated methylated DNA at sites distant from the methylation site. Lane M contains a molecular weight marker (λ phage DNA digested with Hind III).

Finally, we incubated 3 μg of either Hpa II methylated or unmethylated pSVE3 overnight with 26.5 μM CDDP. After removing unbound cDDP and buffer, samples were analyzed for bound platinum by FAAS. Table III shows the increase in platinum bound to methylated DNA compared to unmethylated DNA.

### Discussion

We have attempted to elucidate the mechanism underlying the synergistic cytotoxocity between DAC and cDDP. We first demonstrated that labeled DAC is taken up by CMT3 cells and incorporated into DNA. Surprisingly, we also detected incorporation of DAC into RNA. DAC has been shown to be uniquely incorporated into DNA in human colon carcinoma cells (Glazer & Knode, 1984). However, different cell types are capable to metabolise DAC in different ways. For example the block in colony forming activity, caused by DAC, can be relieved either with cytidine or deoxycytidine in HeLa cells (Snyder & Lachmann, 1989) but only with deoxycytidine in B16 melanoma cells (Cortvrindt *et al.*, 1987) and human leukaemic progenitor cells (Bhalla *et al.*, 1987). In addition, the activity of the enzyme cytidine deaminase, which converts cytidine into uridine, was shown to be increased in HL-60 cells after treatment with DAC (Momparler & Laliberte, 1990). These studies suggest that DAC can be shunted into the RNA pool as we have found.

We showed that in the presence of cDDP, there is decreased DAC uptake by CMT3 cells, ruling out the possibility of synergy at the level of drug entry into the cell; however, this result suggests that cDDP may partially affect cellular transport of nucleosides by crosslinking proteins at the cell surface. The fact that addition of cDDP decreased the incorporation of DAC into RNA may reflect a decrease in transcription caused by cDDP or an increase in RNA degradation.

It seems unlikely that DAC could increase incorporation of cDDP into DNA. It was shown that DAC upregulates the expression of metalothionein (Waalkes *et al.*, 1988) which was also reported as the responsible for the resistance of cells to cDDP, melphalan and chlorambucil (Kelley *et al.*, 1988). Thus, only a decrease in the effectivity of cDDP could have

**Figure 4** Increased sensitivity of Hpa II methylated pSVE3 DNA to cDDP mediated inhibition of DNA replication. CMT3 cells were transfected with nonmethylated (slots 1–6) or Hpa II methylated DNA (Slots 7–12). The cells were untreated (Slots 1, 7) or treated with cDDP 0.5 μM (Slots 2, 8); 1 μM (slots 3, 9); 5 μM (slots 4, 10); 25 μM (slots 5, 11) or 53 μM (Slots 6, 12). Non-chromosomal DNA was extracted and restricted with Msp I. After electrophoresis, the nucleic acids were transferred to a nylon filter and probed with $^{32}$P-labelled pSVE3. Hybridisation to the 3.3 kb SV40 DNA fragment is shown.

**Table III** *In vitro* cDDP binding methylated and non-methylated DNA

| | Ratio cDDP/DNA (ng μg$^{-1}$) | |
|---|---|---|
| | *methylated* | *non-methylated* |
| **exp. 1** | 46.51 | 34.54 |
| **exp. 2** | 53.57 | 32.44 |

Hpa II methylated and non-methylated pSVE3 DNA (3 μg) was incubated *in vitro* with 26.5 μM cDDP. Bound platinum was measured by FAAS (see Methods).

been expected.

We also showed that *in vivo* DAC substituted DNA bound more cDDP than unsubstituted DNA. This result was confirmed by direct measurements of bound platinum by FAAS, after incubating cDDP with DAC-substituted and unsubstituted DNA. These results suggest that the interaction between the two drugs takes place at the DNA level, the role of DAC being to increase the amount of bound cDDP. In these experiments, inhibition of DNA synthesis is the observed outcome of cDDP binding; however, one could predict that gene transcription might also be affected.

We attempted to clarify the role of DAC is this enhancement of cDDP binding. DAC is well know for its role in DNA hypomethylation. During DNA replication, DAC is incorporated as a cytosine analog into the newly synthesised strand. The parent strand remains methylated; however, in the presence of DAC, the action of methyltransferase is inhibited and the daughter strand is therefore unmethylated (Cruesot *et al.*, 1982). Because methylation of a daughter strand requires prior methylation of the parent strand, subsequent round of DNA synthesis after DAC substitution result in generally hypomethylated DNA (Bird, 1978). It would seem possible, then, that DAC-induced hypomethylation might reveal additional cDDP binding sites, particularly in GC-rich regions such as occur frequently in eukaryotic promotors and other regulatory sites. In favour of such suggestion is the observation that increased protein binding to DNA was found at hemymethylated sites after DAC treatment (Michalowsky & Jones, 1987); since cDDP cross-links both proteins and DNA (Ciccarelli *et al.*, 1985) such sites could provide for an excellent substrate for cDDP binding. However, against this explanation is the fact that we were able to demonstrate synergy between DAC and cDDP in a system where further hypomethylation could not occur, i.e. by using a plasmid substrate that was already unmethylated. Yet, the possibility that DAC itself increases protein binding to DNA can not be ruled out.

The lack of enhancement of cDDP binding to hypomethylated DNA was subsequently shown by FAAS, restriction analysis, and *in vivo* DNA replication studies comparing cDDP-treated methylated and unmethylated plasmid DNA. In fact, methylated DNA is found in all three experimental systems to bind more cDDP than unmethylated DNA. This has led us to conclude that the role of DAC in enhancing cDDP binding to DNA is independent of its role as a hypomethylating agent. This is consistent with the results of Frost *et al.*, who were unable to correlate the degree of DAC/cDDP synergy in cytotoxicity assays with the extent of DNA hypomethylation (Abbruzzesse & Frost, 1992; Frost *et al.*, 1990).

The enhancement of cDDP binding to methylated DNA was surprising. Methylation of cytosines is thought to play a role in regulation of gene function (Jones & Taylor, 1980) and transcriptionally active mammalian genomic DNA is frequently hypomethylated (Sanford *et al.*, 1985). Methylation of cytosines in GC-rich sequences in promoter regions may inhibit binding of RNA polymerase or other regulatory proteins, as the methyl group extends into the major groove (Razin & Riggs, 1980). The role of the methyl group may simply be to produce steric hindrance. Alternatively, topologic changes may be involved. Stretches of alternating purines and pyrimidines are known to adopt the unusual left-handed Z DNA conformation (Wang *et al.*, 1982) and this topologic form is stabilised by methylation of cytosines (Behe & Felsenfeld, 1981). Methylation of a critical promotor region may produce or stabilise a DNA conformation that is no longer recognised by the transcriptional machinery.

In a similar manner, the conformation adopted by methylated DNA may actually make it more accessible to cDDP. This would predict that cDDP binding should be enhanced not only at the precise methylation site, but also for a distance upstream and downstream. The restriction studies described in Figures 2 and 3 show exactly this effect. In Figure 2 it is apparent that methylation of Hpa II sites has resulted in enhanced cDDP binding not only to Hpa II but also Hha I sites, as evidenced by incomplete restriction with both enzymes. Conversely, methylation of Hha I sites and treatment with cDDP limits restriction at Hpa II sites (Figure 3). We would propose, then, that certain conformations of DNA are more amenable to cDDP binding, and that one of these conformations is produced when short stretches of repetitive GC sequences are methylated.

In view of the above findings with methylated and unmethylated DNA, it is interesting to speculate about possible mechanisms for the enhancement of cDDP binding to DAC-substituted DNA. It appears that hypomethylation is not necessary for DAC to produce this effect. An alternative suggestion is that DAC incorporation into DNA might induce topologic changes that allow for an increase in cDDP binding, similar to the observation with methylated DNA. Unfortunately, DAC substituted DNA has not been analysed by X-ray crystallography, and therefore direct evidence for DAC-induced DNA conformational changes is lacking. However, some indirect evidence does exist. Jones and Taylor (Jones & Taylor, 1980) have shown than an approximate 5% substitution of 5-azacytidine for cytidine resulted in 80–85% inhibition of cytidine methylation. This suggests that the incorporated azanucleotide produces an effect distant from its immediate surroundings, similar to that which we have seen in our restriction studies using cDDP-treated methylated DNA. Furthermore, DAC-substituted genomic DNA contains fragile sites (Djalali *et al.*, 1990), and is more labile to single stand cleavage in alkali (D'Incalci *et al.*, 1985). Marked alterations in the chromosome structure and condensation patterns of DNA from $GH_1 2C_1$ rat pituitary cells treated with 5-azacytidine have been reported by Parrow *et al.*, (Parrow *et al.*, 1989). Additionally, the free azanucleotide is able to attain different tautomeric forms (Saenger, 1984).

Future studies might be detected towards addressing the interesting possibility that DNA topologic alterations might occur secondary to DAC incorporation. Insight could be gained into the role of this compound as a hypomethylation agent, a differentiation agent, and a cancer chemotherapeutic drug.

The authors thank to Dr Yacov Gluzman for the CMT3 cells and to Dr Yacov Hartman for the pSVE3 plasmid used in this study. This work was supported in part by PHS Grants CA41525 and 39853 and a grant from Pharmachemie, BPV, Haarlem, Netherlands.

### References

ABBRUZZESSE, J.L. & FROST, P. (1992). Studies of the mechanism of the synergistic interaction between 2′deoxy-5azacytidine and cisplatin. *Cancer Chemother. Pharm.*, **30**, 31–36.

BEHE, M. & FELSENFELD, G. (1981). Effects of methylation on a synthetic polynucleotide: The B-Z transition in poly(dG-m$^5$dC)poly(dG-m$^5$dC). *Proc. Natl Acad. Sci. USA*, **78**, 1619–1623.

BHALLA, K., COLE, J., MACLAUGHLIN, W., ARLIN, Z., BAKER, M., GRAHAM, G & GRANT, S. (1987). Effect of deoxycytidine on the metabolism and cytotoxicity of 5-aza-2′-deoxycytidine and arabinosyl 5-azacytosine in normal and leukemic human myeloid progenitor cells. *Leukemia*, **1**, 814–819.

BIRD, A.P. (1978). Use of restriction enzymes to study eukaryotic DNA methylation: II. The symmetry of methylated sites supports semi-conservative copying of the methylation pattern. *J. Mol. Biol.*, **118**, 49–60.

CARADONA, J.P., LIPPARD, S.J., GAIT, M.J. & SINGH, M. (1982). The antitumor drug cis-[Pt($NH_3$)$_2$$Cl_2$] forms an intrastrand d(GpG) cross-link upon reaction with [d(ApGpGpCpCpT)]$_2$. *J. Am. Chem. Soc.*, **104**, 5793–5795.

CHERNAJOVSKY, Y. (1989). Constitutive *in vitro* binding of nuclear proteins to the 5′-flanking region of 6-16, a human gene inducible by $\alpha$, $\beta$ interferons. *FEBS Lett.*, **258**, 323–330.

CHOMCZYNSKI, P. & QASBA, P.K. (1984). Alkaline transfer of DNA to plastic membrane. *Biochem. Biophys. Res. Commun.*, **122**, 340–344.

CICCARELLI, R.B., SOLOMAN, M.J., VARSHAVSKY, A. & LIPPARD, S.J. (1985). *In vivo* effects of cis- and trans-diamminedichloroplatinum (II) on SV40 chromosomes: differential repair, DNA-protein cross-linking, and inhibition of replication. *Biochemistry*, **24**, 7533–7540.

CORTVRINDT, R., BERNHEIM, J., BUYSSENS, N. & ROOBOL, K. (1987) 5-azacytidine and 5-aza-2′-deoxycytidine beahave as different antineoplastic agents in B16 melanoma. *Br. J. Cancer*, **56**, 261–265.

CREUSOT, F., ACS, G.A. & CHRISTMAN, J.K. (1982). Inhibition of DNA methyltransferase and induction of Friend erythroleukemia cell differentiation by 5-azacytidine and 5-aza-2′deoxycytidine. *J. Biol. Chem.*, **257**, 2041–2048.

D'INCALCI, M, COVEY, J.M., ZAHARKO, D.S. & KOHN, K.W. (1985). DNA alkali-labile sites induced by incorporation of 5-aza-2′-deoxycytidine into DNA of mouse leukemia L1210 cells. *Cancer Res.*, **45**, 3197–3202.

DJALALI, M., ADOLPH, S., STEINBACH, H., WINKING, H. & HAMEISTER, H. (1990). Fragile sites induced by 5-azacytidine and 5-azadeoxycytidine in the murine genome. *Hereditas*, **112**, 77–81.

FEINBERG, A.P. & VOGELSTEIN, B. (1983). A technique for radiolabeling DNA restriction endonuclease fragments to high specific activity. *Anal. Biochem.*, **132**, 6–13.

FROST, P., ABBRUZZESE, J.L., HUNT, B., LEE, D. & ELLIS, M. (1990). Synergistic cytotoxicity using 2′-deoxy-5-azacytidine and cisplatin or 4-hydroperoxycyclophosphamide with human tumours. *Cancer Res.*, **50**, 4572–4577.

GERARD, R.D. & GLUZMAN, Y. (1985). New host cell system for regulated Simian Virus 40 DNA replication. *Mol. Cell. Biol.*, **5**, 3231–3240.

GLAZER, R.I. & KNODE, M.C. (1984). 1-b-D Arabinosyl-azacytosine: Cytocidal activity and effects on the synthesis and methylation of DNA in human colon carcinoma cells. *Mol. Pharmacol.*, **26**, 381–387.

KELLEY, S.L., BASU, A., TEICHER, B.A., HACKER, M.P., HAMER, D.H. & LAZO, J.S. (1988). Overexpression of metallothionein confers resistance to anticancer drugs. *Science*, **241**, 1813–1815.

HANAHAN, D. (1983). Studies on transformation of *E. coli* with plasmids. *J. Mol. Biol.*, **166**, 557–580.

HARTMAN, J.R., NAYAK, D.P. & FAREED, G.C. (1982). Human influenza virus hemagglutinin is expressed in monkey cells using simian virus 40 vectors. *Proc. Natl Acad. Sci. USA*, **79**, 233–237.

HIRT, B. (1967). Selective extraction of polyoma DNA from infected mouse cell cultures. *J. Mol. Biol.*, **26**, 365–369.

JONES, P.A. & TAYLOR, S.M. (1980). Cellular differentiation, cytidine analogs and DNA methylation. *Cell*, **20**, 85–93.

MICHAELOWSKY, L.A. & JONES, P.A. (1987). Differential nuclear protein binding to 5-aza-cytosine-containing DNA as a potential mechanism for 5-aza-2′-deoxycytidine resistance. *Mol. Biol.*, **7**, 3076–3083.

MOMPARLER, R.L. & LALIBERTE, J. (1990). Induction of cytidine deaminase in HL-60 myeloid leukemic cells by 5-aza-2′-deoxycytidine. *Leuk. Res.*, **14**, 751–754.

NEWMAN, R.A., KHOKHAR, A.R., SUNDERLAND, B.A., TRAVIS, E.L. & BULGER, R.E. (1986). A comparison in rodents of renal and intestinal toxicity of cisplatin and a new water-soluble antitumor platinum complex: N-methyliminodiacetato-diaminocyclohexane platinum (II). *Toxicol, Appli. Pharmacol.*, **84**, 454–463.

PARROW, V.C., ALESTROM, P. & GAUTVIK, K.M. (1989). 5-Azacytidine-induced alterations in the $GH_1 2C_1$ cells effects on cellular morphology, chromosome structure, DNA and protein synthesis. *J. Cell. Sci.*, **93**, 533–543.

PINTO, A.L. & LIPPARD, S.J. (1985). Binding of the antitumor drug cir-diamminedichloroplatinum (II) (Cisplatin) to DNA. *Biochim. Biophys. Acta.*, **780**, 167–180.

RAZIN, A. & RIGGS, A.D. (1980). DNA methylation and gene function. *Science*, **210**, 604–610.

SAMBROOK, J., FRITSCH, E.F. & MANIATIS, T. (1989). *Molecular Cloning. A Laboratory Manual.* 2nd Ed. Cold Spring Harbor Laboratory Press. pp. 2.79–2.81.

SAENGER, W. (1984). *Principles of Nucleic Acid Structure.* p.189. Springer Verlag, N.Y.

SANFORD, J.P., CHAPMAN, V.M. & ROSSANT, J. (1985). DNA methylation in extraembryonic lineages of mammals. *Trends in Genetics*, **1**, 89–93.

SNYDER, R.D. & LACHMANN, P.J. (1989). Differential effects of 5-aza-cytidine and 5-aza-deoxycytidine on cytotoxicity, DNA strand breaking and repair of X-ray-induced DNA damage in Hela cells. *Mutat, Res.*, **226**, 185–190.

SOUTHERN, E. (1975). Detection of specific sequences among DNA fragments separated by gel elctrophoresis. *J. Mol. Biol.*, **98**, 503–517.

USHAY, H.M., TULLIUS, T.D., LIPPARD, S.J. (1981). Inhibition of the BamHI cleavage and unwinding of pBR322 deoxyribonucleic acid by the antitumor drug cis-dichlorodiammineplatinum (II). *Biochemistry*, **20**, 3744–3748.

VESELY, J. & CIHAK, A. (1977). Incorporation of a potent antileukemic agent, 5-aza-2′-deoxycytidine, into DNA of cells from leukemic mice. *Cancer Res.*, **37**, 3684–3689.

WAALKES, M.P., MILLER, M.S., WILSON, M.J., BARE, R.M. & MCDOWELL, A.E. (1988). Increased metallothionein gene expression in 5-aza-2′-deoxycytidine-induced resistance to cadmium cytotoxicity. *Chem. Biol. Interact.*, **66**, 189–204.

WANG, A.H., FUJII, S., VAN BOOM, J.H. & RICH, A. (1982). Right-handed and left-handed double-helical DNA: structural studies. *Cold Spring Harbor Symp. of Quant. Biol.*, Vol. XLVII (1): 33–44.