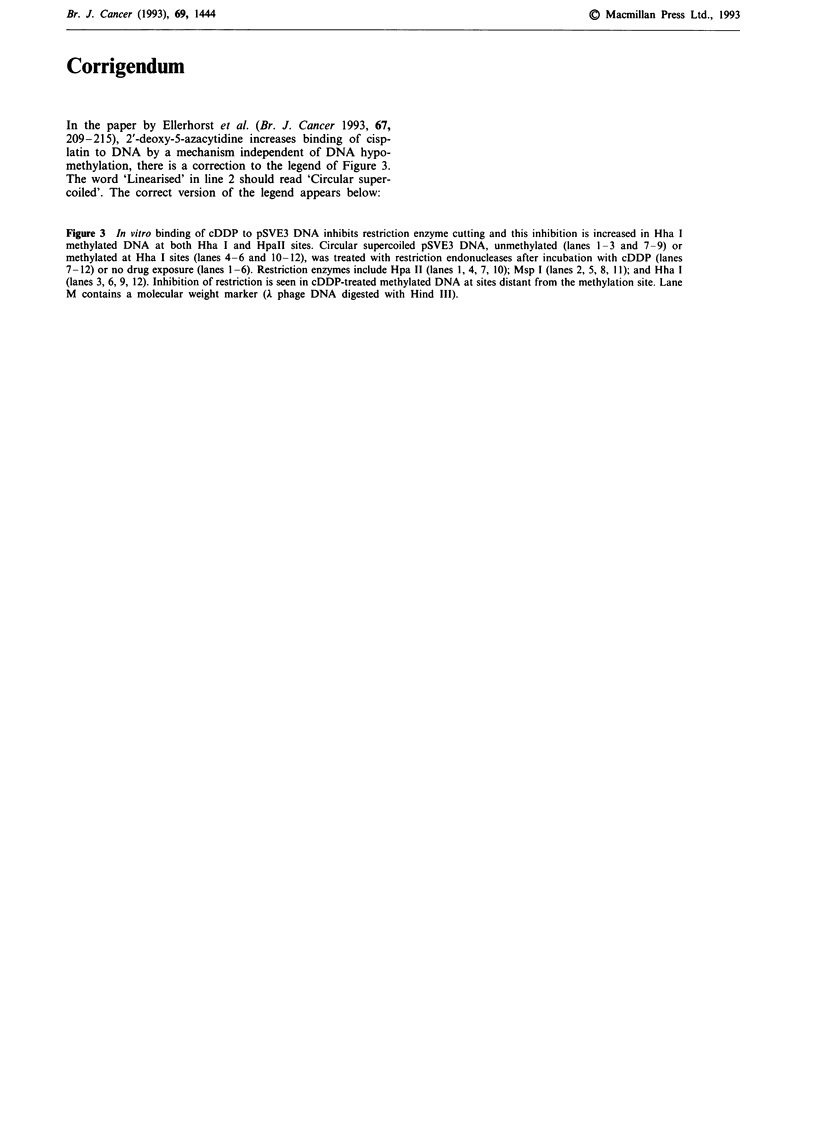# Corrigendum

**Published:** 1993-06

**Authors:** 


 © Macmillan Press Ltd., 1993

### Corrigendum

In the paper by Ellerhorst *et al.* (*Br. J. Cancer* 1993, **67**, 209–215), 2′-deoxy-5-azacytidine increases binding of cisplatin to DNA by a mechanism independent of DNA hypomethylation, there is a correction to the legend of Figure 3. The word 'Linearised' in line 2 should read 'Circular supercoiled'. The correct version of the legend appears below:

**Figure 3** *In vitro* binding of cDDP to pSVE3 DNA inhibits restriction enzyme cutting and this inhibition is increased in Hha I methylated DNA at both Hha I and HpaII sites. Circular supercoiled pSVE3 DNA, unmethylated (lanes 1–3 and 7–9) or methylated at Hha I sites (lanes 4–6 and 10–12), was treated with restriction endonucleases after incubation with cDDP (lanes 7–12) or no drug exposure (lanes 1–6). Restriction enzymes include Hpa II (lanes 1, 4, 7, 10); Msp I (lanes 2, 5, 8, 11); and Hha I (lanes 3, 6, 9, 12). Inhibition of restriction is seen in cDDP-treated methylated DNA at sites distant from the methylation site. Lane M contains a molecular weight marker ($\lambda$ phage DNA digested with Hind III).